# Editorial: Emerging artificial intelligence technologies for neurological and neuropsychiatric research

**DOI:** 10.3389/fnins.2024.1518442

**Published:** 2024-11-14

**Authors:** Ahmed Elnakib, Fahmi Khalifa, Ahmed Soliman, Ahmed Shalaby, Mostafa Elhosseini

**Affiliations:** ^1^Electrical and Computer Engineering Department, School of Engineering, Penn State Erie-The Behrend College, Erie, PA, United States; ^2^Electronics and Communication Engineering, Mansoura University, Mansoura, Egypt; ^3^Electrical and Computer Engineering, Morgan State University, Baltimore, MD, United States; ^4^Department of Computers and Control Systems Engineering, Faculty of Engineering, Mansoura University, Mansoura, Egypt; ^5^Department of Bioinformatics, University of Texas Southwestern Medical Center, Dallas, TX, United States; ^6^College of Computer Science and Engineering, Taibah University, Yanbu, Saudi Arabia

**Keywords:** artificial intelligence, machine learning, neurological disorders, neuroimaging, cognitive assessment

## Introduction

The advent of artificial intelligence (AI) has revolutionized neurological and neuropsychiatric research, offering powerful and efficient tools to analyze complex, multiomodal, and vast datasets that were previously intractable. Recent advancement of integrated Machine learning (ML) and deep learning (DL) algorithms have enabled researchers to uncover patterns and novel insights, advancing our understanding of neural mechanisms and wide range of disorders. This editorial introduces four recent research contributions that exemplify the transformative application of emerging AI-based technologies in this field, highlighting their substantial impact on diagnosis, treatment, and patient care. The finding of these advancements, and future work, will be an integral part of the future of personalized medicine, pushing the boundaries of what is possible in neurological and neuropsychiatric care.

## Overview of the contributing articles

Paper 1: *Identifying major predictors for parenting stress in a caregiver of autism spectrum disorder using machine learning models* by Choi et al.

This study employs explainable ML models to identify key predictors of parenting stress and its subclasses among caregivers of children with autism spectrum disorder (ASD). The study analyzed data from 496 participants and utilized four different ML classifiers [eXtreme gradient boosting (XGBoost), support vector machine (SVM), logistic regression, and random forest (RF)]. RF outperformed other classifiers for predicting parental distress, difficult child, and total parenting stress with area under the curve (AUC) values of 0.83, 0.81, and 0.86, respectively, while SVM outperformed the rest for predicting parent-child dysfunctional interaction with AUC value of 0.81. Significant predictors included child-related factors like aggressive behavior and anxiety/depression, as well as caregiver-related factors such as depression and social introversion. The top five significant predictors for each subclass are listed in this study. The use of interpretable models offers valuable insights for clinicians to identify at-risk caregivers and provide timely interventions, ultimately improving outcomes for families affected by ASD.

Paper 2: *A review of the applications of generative adversarial networks to structural and functional MRI based diagnostic classification of brain disorders* by Huynh and Deshpande

The second study provides a comprehensive review of the recent applications of generative models, particularly generative adversarial networks (GANs) in enhancing diagnostic classification of brain disorders using structural and functional MRI data. GANs effectively tackle challenges such as data scarcity and class imbalance by generating high-quality synthetic data to augment existing datasets, thereby significantly improving model robustness and performance. The review discusses the latest advancements in GAN architectures and highlights the need for standardized metrics to assess the quality of generated data. The authors suggest integrating GANs with graph convolutional networks (GCNs) and self-attention mechanisms to better capture complex brain network features, further enhancing diagnostic precision. They emphasize the importance of collaboration between method developers and end-users to tailor GAN applications effectively in real-world challenges in neuroimaging.

Paper 3: *The identification of cognitive impairment in Parkinson's disease using biofluids, neuroimaging, and artificial intelligence* by Dennis and Strafella

The third study explores the use of AI to identify cognitive impairment in Parkinson's disease (PD) patients. The authors conduct an in-depth analysis of both biofluids and neuroimaging data to enhance the precision and reliability of cognitive impairment detection. Reviewing literature that applies ML algorithms to different biomarkers, from structural and functional MRI scans, positron emission tomography (PET), beta-amyloid-42, tau proteins, the authors found that combining biofluid and imaging biomarkers enhances diagnostic accuracy, with some studies reporting AUC values above 0.90. The paper underscores the importance of proper model development protocols, noting that studies with rigorous methodologies tend to report higher capabilities. The findings suggest that integrating multiple biomarker types and adhering to robust training and testing procedures are crucial for developing reliable diagnostic tools.

Paper 4: *Objective assessment of cognitive fatigue: a bibliometric analysis* by Han et al.

The fourth study presents a bibliometric analysis of research on the objective assessment of cognitive fatigue spanning from 2007 to 2023. Analyzing 1,094 articles, the study identifies key trends, influential contributors, and emerging hotspots; thus, offers a detailed map of the field's evolution. Modalities such as electroencephalogram (EEG), functional near-infrared spectroscopy (fNIRS), heart rate variability (HRV), electrooculography (EOG), and eye-tracking are highlighted as central tools for objective fatigue assessment. Additionally, the analysis reveals a growing interest in developing innovative deep neural networks and multimodal fusion techniques to significantly enhance detection accuracy. Future research is expected to focus on practical applications like monitoring fatigue in drivers and pilots, emphasizing the societal relevance ad real-world relevance of these advancements.

## Emerging trends and technologies

Collectively, these articles highlight several emerging trends:

- Explainable and interpretable AI models for precision diagnosis: There is a shift toward using interpretable AI/ML models, enabling clinicians to understand and trust AI predictions. This is crucial for integrating AI tools into clinical practice.
- Data augmentation with generative AI: Generative models, such as GANs, offer solutions to data scarcity by generating high-quality synthetic data, enhancing model training, and addressing class imbalance in medical datasets.
- Multimodal biomarker integration: Combining different types of biomarkers (e.g., neuroimaging and biofluids) improves diagnostic accuracy by providing a more comprehensive view of the patient's condition.
- Practical and societal applications: Toward real-world, impactful applications of AI technologies, the contributing articles to this issue also emphasize on the growing need on developing monitoring tools for high-risk assessments.
- Advanced neural networks and multimodal fusion: The development of sophisticated neural networks and the fusion of multiple data modalities enhance the assessment of cognitive states, such as fatigue, leading to more accurate and reliable tools.

## Challenges and future directions

Despite significant progress, several challenges remain:

- Data quality and standardization: Ensuring high-quality data and developing standardized evaluation metrics are critical. Inconsistent data quality and data imbalance can hinder model performance and generalizability.
- Interpretability and trust: Complex AI models can be opaque, making it difficult for clinicians to interpret results. Emphasizing explainable AI is essential for clinical acceptance.
- Multimodal integration: Integrating various data sources necessitate advanced and novel data fusion algorithm. This in turn can complicate model development, increase the risk of overfitting, and the processing time.
- Interdisciplinary collaboration: Effective application of AI in neurology requires collaboration between AI specialists, neuroscientists, and clinicians to address practical clinical needs and ensure models are both technically sound and clinically relevant.
- Ethical considerations: Issues related to data privacy, consent, and algorithmic bias must be addressed to ethically implement AI technologies in healthcare.

Future research should focus on:

- Enhancing model robustness: Developing models that generalize across diverse populations and settings, possibly by incorporating longitudinal data and testing on real-world imbalanced datasets.
- Integrating advanced techniques: Exploring the combination of GANs with GCNs and self-attention mechanisms to improve the capture of complex neural patterns.
- Practical applications: Applying AI advancements to real-world scenarios, such as fatigue monitoring in transportation and occupational settings, to address pressing societal challenges.

## Conclusion

The contributions in this special issue demonstrate the transformative potential of AI technologies in advancing neurological and neuropsychiatric research. By addressing complexities in data analysis, multi-information fusion, enhancing diagnostic accuracy, and providing objective assessments, these studies contribute valuable tools for improving patient care. The emerging trends emphasize the importance of explainable AI, data augmentation, multimodal integration, and advanced neural networks.

Moving forward, interdisciplinary collaboration and adherence to ethical standards will be essential in harnessing AI's full potential. By building on these foundations, future research can explore new frontiers, ultimately bridging the gap between technological innovation and real-world clinical application. The integration of AI into neurological research holds great promise for better understanding neural disorders and developing effective interventions that enhance patient outcomes.

